# Different storage times and their effect on the bending load to failure testing of murine bone tissue

**DOI:** 10.1038/s41598-020-74498-8

**Published:** 2020-10-15

**Authors:** Thomas M. Tiefenboeck, Stephan Payr, Olga Bajenov, Theresia Dangl, Thomas Koch, Micha Komjati, Kambiz Sarahrudi

**Affiliations:** 1 Department of Orthopaedics and Trauma Surgery, Division of Trauma Surgery, Medical University of Vienna, Waehringerguertel 18-20, 1090 Vienna, Austria; 2 Institute of Materials Science and Technology, TU Wien, Vienna, Austria; 3 Department of Orthopaedics, Sacred Heart Hospital of Vienna, Vienna, Austria; 4 Department of Trauma Surgery, Landesklinikum Wiener, Neustadt, Austria

**Keywords:** Tissues, Mechanical properties

## Abstract

Cryopreservation is a well-established method for bone storage. However, the ideal timing of mechanical testing after sacrificing the experimental animals is still under discussion and of significant importance to the presentation of accurate results. Therefore, the aim of this study was to investigate and compare different cryopreservation durations to native murine bone and whether there was an influence on mechanical bone testing. For this study the tibias of 57 female C57BL/6 mice—18-weeks of age—were harvested and randomly allocated to one of four groups with varying storage times: (1) frozen at −80 °C for 3 months, (2) frozen at −80 °C for 6 months, (3) frozen at −80 °C for 12 months and (4) native group. The native group was immediately tested after harvesting. The comparison of the mean strength and load to failure rates demonstrated a significant difference between the storage groups compared to the native control (*p* = 0.007). However, there was no difference in the strength and the load to failure values of bones of all storage groups when compared against each other. Once cryopreservation at −80 °C is performed, no differences of mechanical bone properties are seen up to 12 months of storage. When actual in vivo data is of close interest, immediate testing should be considered and is preferred. If comparison of groups is required and long-time storage is necessary, cryopreservation seems to be an accurate method at present.

## Introduction

Bone storage is an important step in the majority of experiments on bone. Hence, understanding the effects of bone storage is of great importance for orthopaedic and trauma surgery. Freezing is mainly presented as the preferred method for bone preserving as low temperatures slow down the biological and biomechanical processes or even stop these completely^1,2^. Decreasing the metabolic rate by freezing is an important method for conservation and storage. Cryopreservation has been extensively studied as a viable solution to the long-term storage of various biomaterials, like oocytes^3^, stem cells^4,5^, vascular tissues^6^, and even embryos^7^. Also, in animal model’s cryopreservation is presented as a daily routine before final testing. Especially mouse models have been proven to be beneficial for mechanical bone testing. Because of the fact that the biomechanical test is usually performed as the final procedure at the end of experiments, long periods of bone storage may often be required^8^. The long storage time can cause an alteration of the biomechanical properties of bone, therefore, the type of storage method needs to be as accurate as possible^8^.

Due to limited number of the tested animals it is important to choose the right testing procedure as well as the correct storage method. The three-point bending test is described as one of the most appropriate methods to test load to failure in literature^9^.

For the purpose of long-term bone storage, several methods have been described in the literature. We have recently^10^ reported that cryopreservation may be more suitable for long-term storage than other methods, such as using paraformaldehyde or formalin. Ethanol for example leads to an extensive damage to the triple-helical structure of collagen depending on temperature and storage time^11^.

It is known that alcohol or formalin fixation changes the plastic mechanical properties of bone and therefore the use of fresh-frozen bone specimens is recommended in biomechanical studies investigating failure loads^12^. Cryopreservation is also the method of choice in the preservation of bone, meniscal tissue^13^ and tendons^14^ for human use, with preservation times of more than 5 years^15,16^. Cryopreservation for example is the only method that preserves fresh meniscus architectural specificities^13^.

In literature, cryopreservation protocols vary from −4 to −80 °C for various periods but in the majority of cases, freezing and storing the samples at −20 °C is described^17,18^. However, in a recent study by Cheng et al.^15^ −80° was proven to present the lowest effect on mechanical properties even after long-term storage. It is known that different periods of storage lead to changes in bone microarchitecture and therefore change mechanical properties, resulting in misleading results in mechanical testing. However, there is little literature available investigating the time period of cryopreservation of murine bone samples and its influence on mechanical properties.

Therefore, the aim of this study was to investigate the influence of cryopreservation periods on murine bone tissue compared to native tissue and the impact it has on mechanical bone testing.

We hypothesized that there is a difference between the load to failure rates with regard to different storage times.

## Results

**Figure 1:**
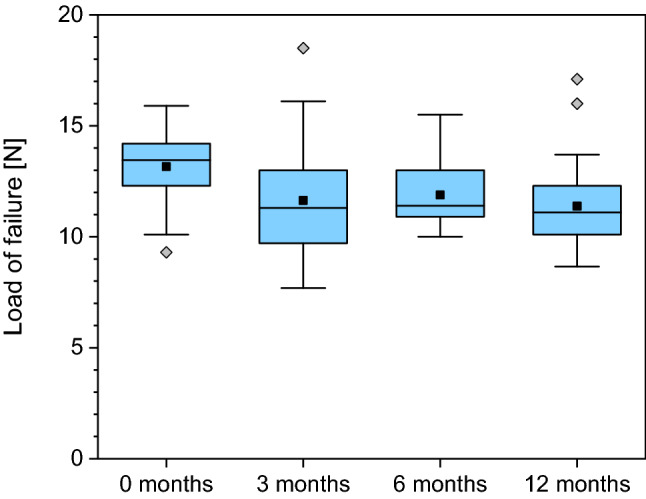
Boxplot presenting the load to failure regarding the four groups.

Significant changes (*p* = 0.007) were found when comparing the load to failure between the native samples and the frozen samples regardless of the freezing duration. Comparing the 3, 6- and 12-months freezing groups there were no significant differences in the load to failure. The mean load to failure in the native group was 13.2 N (median 13.5 N; range 9.3 to 15.9 N; STD 1.5 N). The 3-month group showed a mean load to failure of 11.2 N (median 11.1 N; range 8.7 to 17.1 N; STD 1.8 N). The 6-month group showed a mean load to failure of 11.9 N (median 11.4 N; range 10 to 15.5 N; STD 1.5 N). The 12-month group showed a mean load to failure of 11.4 N (median 11.1 N; range 4.6 to 18.5 N; STD 2.7 N) (Fig. 1).

No significant difference was found with regards to the mean mouse weight between all groups (native 27.3 g; 3 months freezing 27.1 g; 6 months freezing 27.1 g; 12 months freezing 27.9 g).

**Figure 2:**
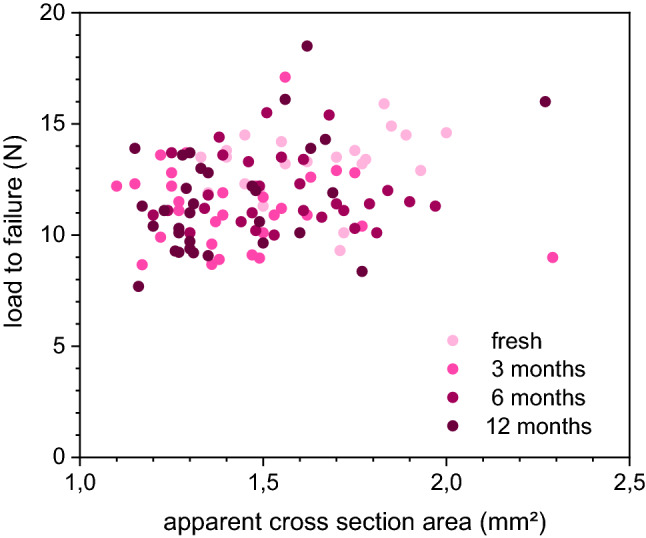
Details of apparent cross-sectional area regarding the load to failure.

**Figure 3:**
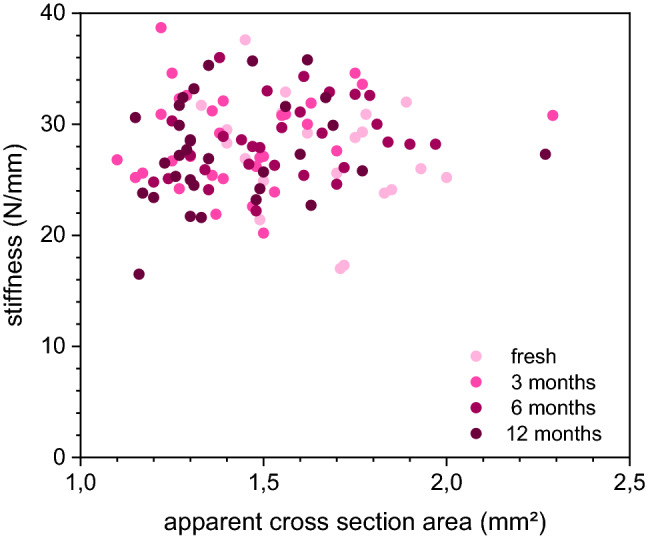
Details of apparent cross-sectional area regarding the stiffness.

The mean length of the harvested samples was 18 mm, without showing significant differences in mean length between the harvested tibias in the groups (native 18.7 mm vs. 3 months freezing 18.3 mm vs. 6 months freezing 18.8 mm vs. 12 months freezing 18.2 mm). Also, there were no differences found regarding the diameters measured at the area of interest as well as of the cross-sectional area. (Figs. 2 and 3).

There was no significant difference between mean stiffness (native 27.3 N/mm vs. 3 month freezing 28.7 N/mm vs. 6 months freezing 28.6 N/mm vs. 12 months freezing 27.5 N/mm). A detailed overview is presented in Table 1.

**Table 1:** Details of stiffness, strength, cross sectional area and load to failure of testes bones.

Group	Stiffness in N/mm	Cross sectional area in mm^2^	Load to failure in N
Native bone (n = 22)	27.3 (27.6; 17 to 37.6; ± 4.8)	1.4 (1.4; 1.1 to 1.8; ± 0.2)	13.2 (13.5; 9.3 to 15.9; ± 1.5)
Freezing 3 months (n = 33)	28.6 (27.6; 20.2 to 38.7; ± 4.2)	1.4 (1.3; 1 to 2.1, ± 0.3)	11.4 (11.1; 8.7 to 17.1; ± 1.9)
Freezing 6 months (n = 29)	28.6 (28.2; 22.2 to 36; ± 3.3)	1.5 (1.5; 1.2 to 2.6; ± 0.3)	11.9 (11.4; 10 to 15.5; ± 1.5)
Freezing 12 months (n = 33)	27.6 (27.3; 16.5 to 35.8; ± 4.5)	1.3 (1.3; 0.9 to 2.8; ± 0.4)	11.6 (11.3; 7.7 to 18.5; ± 2.4)

N—Newton.

**Figure 4:**
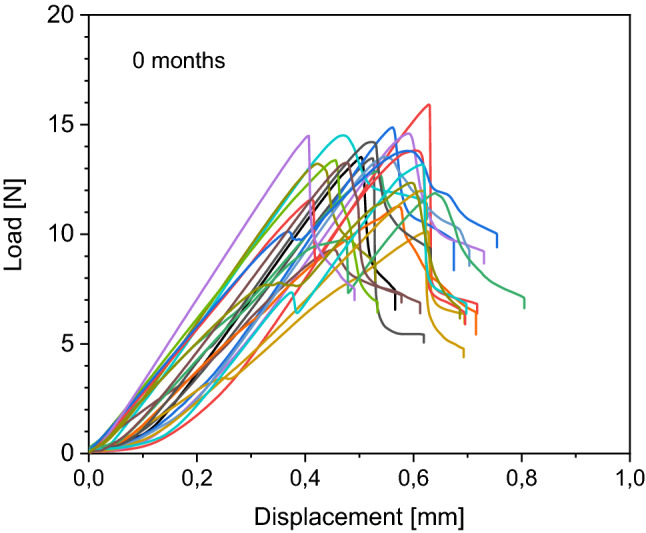
Behaviour of the native samples during load to failure test. Each curve represents one sample during the load to failure test. Starting at 0 N, load increases to maximum and then decreases.

**Figure 5:**
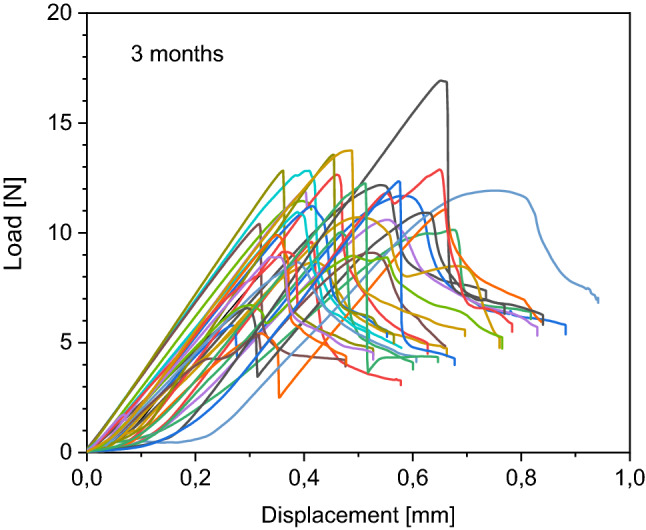
Behaviour of the 3 months frozen samples during load to failure test. Each curve represents one sample during the load to failure test. Starting at 0 N, load increases to maximum and then decreases.

**Figure 6:**
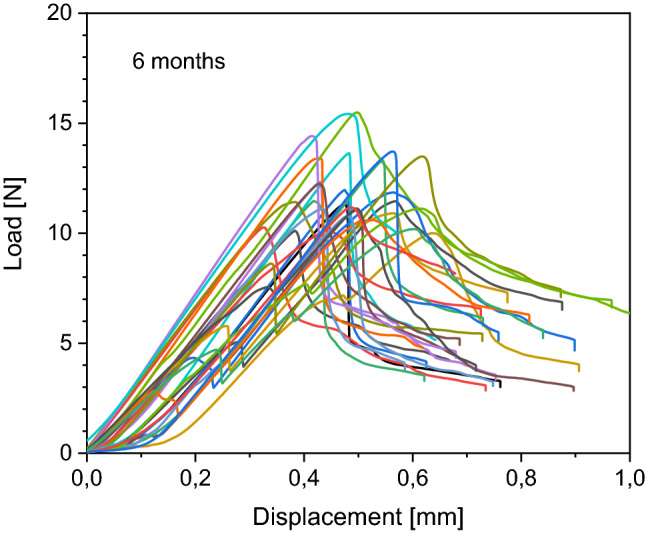
Behaviour of the 6 months frozen samples during load to failure test. Each curve represents one sample during the load to failure test. Starting at 0 N, load increases to maximum and then decreases.

**Figure 7:**
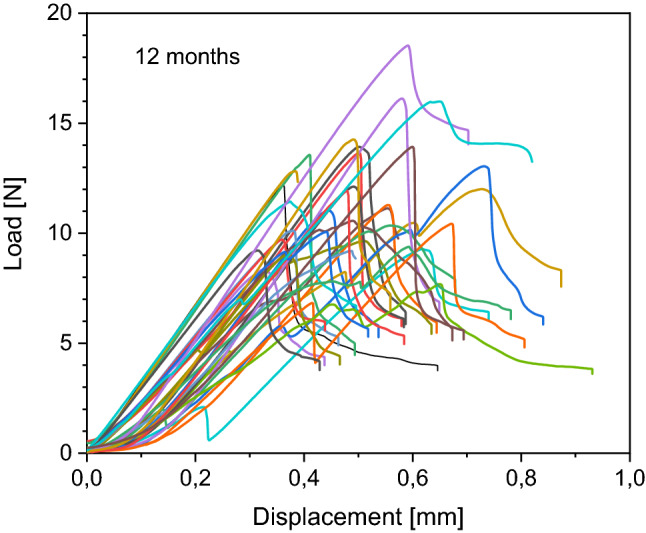
Behaviour of the 12 months frozen samples during load to failure test. Each curve represents one sample during the load to failure test. Starting at 0 N, load increases to maximum and then decreases.

Details of load to failure curves of each bone are demonstrated in Figs. 4, 5, 6 and 7. All fractures occurred in the before mentioned defined area of interest.

## Discussion

This study is one of the first studies investigating different periods of cryopreservation in murine bone tissue. The storage of murine bone tissue is essential for the implementation of biomechanical testing. Often it is necessary to store the harvested bone for a longer time till testing is possible. This is due to the high volume of samples, pragmatic availability of equipment to perform load to failure tests and even general pragmatic lab considerations. Therefore, it is essential that the used method is able to provide reproduceable biomechanics and histological workup. For biomechanical testing, load to failure tests are most commonly used and the three-point bending test represents one of the most appropriate methods^9^.

Despite the ongoing efforts and discussions of the biomechanical community the scientific literature has yet to unify a common methodological approach to long-term bone storage. Different protocols are presented in literature including storage with ethanol, formaldehyde, paraformaldehyde and cryopreservation^17, 18^. These different storage methods each present with advantages and disadvantages. In a recent paper, our group could demonstrate that short-term storage of murine tibial bone tissue do not affect the load to failure, independent of the used method^10^. The lack of short-term detrimental effects as evidenced in our murine study^10^ is supported by the findings of Beaupied et al.^18^ who showed that one month storage in alcohol or deep-freezing seemed to induce no harmful effect on densitometric, microarchitectural and biomechanical parameters of rat femurs.

However, there are only a few studies present in literature dealing with long-term storage and the influence of different time spans on tissue properties. Therefore, this study aims to close this gap.

Cryopreservation at −80 °C is a simple and easy storage method, however, compared to native bone tissue there is a significant decrease of the load to failure rate. Nevertheless, it is of great importance to point out that in our study there is no significant difference between 3, 6 or 12 months of cryopreservation regarding the load to failure in the three-point bending test. Another important point is that frozen bone samples can also be taken for histological preparation and analysis^19^, which makes it unique compared to other methods, such as alcohol fixation. Concluding from this, it seems that cryopreservation of bone tissue is ideal for studies investigating load to failure of bone when long-term storage is needed.

In literature, cryopreservation protocols vary from −4 to −80 °C for various periods but in the majority of cases, freezing and storing the samples at −20 °C is described. In a study by Cheng et al.^15^ a significant decrease of the elastic modulus and deflection could be shown in the 4% paraformaldehyde group. The maximum load and elastic modulus of the samples in all storage groups were significantly reduced after one week of storage. However, the mechanical properties were close to the fresh control group in the −20° group stored for 2 months. The maximum load presented reduced after 6 months. However, mechanical properties, such as elastic load, maximum load and elastic modulus, were not changed obviously in the −80° storage group. So they concluded that, −80 cryopreservation had little influence on the mechanical properties of bone tissues, which proved that the temperature −80 is a suitable one for long-term preservation^15^. This corresponds with our data, that −80° is suitable for long-term storage, although native bone samples still presents the best to get tested.

So the ideal way of testing these specimens would be to use a frozen control group to rule out the differences between the native and the deep frozen samples regarding the load to failure. Overall the time of freezing (> 3 months) does not have an effect on load to failure in a three-point bending test, which is supported by a study with short-term results showing that freezing has no influence on the mechanical properties of the bovine cortical bone.

The results of this study show that is does make a difference if murine bone tissues are tested immediately or after storage, but these findings can be corrected if using a control group as mentioned before. It also leads to the assumption that the stiffness of bone tissue declines over time when cryopreserved. These findings do not only have consequences for experimental studies, it raises the question if deep frozen allografts might show impaired biomechanical properties compromising fracture stability when used clinically compared to fresh autologous bone grafts. The availability of allografts when a huge bone defect has to be treated is a great enrichment but in current literature there were no studies found addressing this question.

In contrast to our findings, cryopreservation of bone allografts is described in the literature between 6 months to 5 years and even longer without impairments on biomechanics^15,16^. Cryopreservation is used also as the method of choice in the preservation of meniscal tissue^13^ and tendons^14^. However, it should be mentioned when using these tissues, a longer period of ingrowth is needed for integration into the organism. The longer ingrowth period provides a longer period of recovery post-thaw which may mitigate impacted biological function. This might also explain the problem of early implant failure.

## Limitations

This study was limited to murine bone tissue, focusing only on the evaluation of the load to failure rate in a three-point bending test between different storage times. It also needs to be pointed out that a three-point bending test does not entirely cover all biomechanical properties of the bone. Due to the fact that only one biomechanical test is possible it was decided to use the most meaningful test. However, this is one of the first studies comparing a three-point bending test for load to failure of murine bone tissue after −80 °C cryopreservation with different time periods to instantly tested native bone tissue.

It needs to be mentioned that no evaluation of bone quality was made prior to the final load to failure test. The same bone quality across all mice was assumed due to the following factors: same race (C57BL/6), same age and weight of the mice, as well as the same holding and feeding conditions.

## Conclusion

Once cryopreservation at −80 °C has been performed, no differences of mechanical bone properties were seen up to 12 months of storage. When actual in vivo data is of close interest, immediate testing should be considered. If comparison of groups is required and long-term storage is necessary, cryopreservation seems to be an accurate method at present.

## Material and methods

### Animals

This study was performed as a basic research project on murine bone tissue at the Department of Trauma and Orthopedic Surgery, Medical University of Vienna in cooperation with the Institute of Material Science and Technology, Vienna University of Technology.

Sixty female C57BL/6 mice (18 weeks old) were included in this study, all of them serving as a control in another animal experiment. In all mice, except the native ones, the same surgical procedure was performed. All mice were anesthetized with 0.1 ml/10 g narcotic mix (ketamine 0.5 ml + 0.15 ml Rompun + 0.1 ml Dormicum in 5 ml NaCl) via subcutaneous injection with a 27-G needle. Pre-operatively, as well as on the first post-operative day, Enrofloxazin (7.5 mg/kg) was given for infection prophylaxis. Immediately after the surgery 0.1 ml/10 g of glucose mix was injected and all mice received Buprenorphin 0.1 mg/kg s.c. for post-operative analgesic therapy (under general anaesthesia). Animals also received Piritramid 15 mg in 250 ml drinking water ad libitum together with 10 ml glucose (10%) for 5 days. The mice, 5 per group, were held in type 3 Makrolon cages at a temperature of 22 ± 2 °C, humidity of 55% ± 10%, a 12 h light cycle and they received food and water ad libitum.

All animals were sacrificed with ketamine and heart puncture according to the guidelines of the Centre of Biomedical Research of the Medical University of Vienna.

Following this, both tibias were harvested via a direct incision over the bone and separated from the surrounding soft tissue. Additionally, the fibula was dissected from the tibia. The tibias were then randomly divided into four groups with the following storage periods: Group1 frozen at −80 °C for 3 months (n = 31), Group 2 frozen at −80 °C for 6 months (n = 29), Group 3 frozen at −80 °C for 12 months (n = 33) and native group (n = 22). Each of the harvested tibias was individually stored in a plastic tube.

### Storage of bone

In group 1 the bones were stored in a freezer at −80 °C for 3 months. Prior to testing they were defrosted over 72 h in a refrigerator at 4 °C.

In group 2 the bones were stored in a freezer at −80 °C for 6 months. Prior to testing they were defrosted over 72 h in a refrigerator at 4 °C.

In group 3 the bones were stored in a freezer at −80 °C for 12 months. Prior to testing they were defrosted over 72 h in a refrigerator at 4 °C.

In group 4 the bones were referred to as “native group”, these bones were tested directly after harvesting without any storage.

### Biomechanical Testing

The biomechanical testing was performed in accordance to the guidelines of the Institute of Material Science and Technology of the Technical University of Vienna and also took place there. These are in accordance to the testing procedures presented in the literature^9^. To test bone mechanics, load to failure tests are the method of choice – therefore there is a need to decide which kind of test will be used. It was decided to use a three-point bending test, which is described in literature to be one of the appropriate tests to test load to failure^9^. To consider the different cross-sections of the investigated bones, from every bone in the region of interest the largest diameter and the related perpendicular diameter was measured using a digital caliper. Now, it was simplified assumed that the shape of the perimeter follows an ellipse with the largest and the perpendicular diameter as the major and minor axis respectively. From that simplified elliptic shape an apparent cross-section area was calculated.

A total of 115 out of 120 tibial bones underwent a standardized testing procedure. Each bone was mounted on a universal material testing machine (Zwick Z050), equipped with a 100 N load cell. For documentation purposes photographs were taken with a Nikon D500 digital single-lens reflex (DSLR) camera.

The bones were fixed onto the adapter of the material testing system to perform a three-point bending test on the bone^9^.

Final testing was performed in accordance to the standard presented in literature, with a load to failure test^20^.

This study focused on the load to failure values of murine bone samples after different storage periods.

### Statistics

Normal distribution was tested using the Shapiro–Wilk’s test. Homogeneity was evaluated with the Levene test. A mixed-model ANOVA was used to test differences between the four groups. Descriptive statistics (means and standard deviation) were performed for all four groups. Statistical significance was set at a level of *p* < 0.05. Microsoft Excel, SPSS software (Version 25.0, SPSS Inc., Chicago, IL, USA) were used for statistical evaluation.

### Ethics

This study was conducted after postive vote of the animal ethics review board (Ethik-Kommission der MUW zur Beratung und Begutachtung von Forschungsprojekten am Tier) and the BMFWF (Bundesministerium für Wissenschaft, Forschung und Wirtschaft) (ZI. 177/115–97/98 out 2014/15). The ARRIVE guidelines were used and followed during entire duration of the trial.

## Data Availability

The data that support the findings of this study are available from the corresponding author upon reasonable request.
